# ***Barucynips panamensis***, a new genus and species of oak gallwasps (Hymenoptera, Cynipidae, Cynipini) from Panama, and description of one new species of ***Coffeikokkos***

**DOI:** 10.3897/zookeys.277.3942

**Published:** 2013-03-15

**Authors:** Enrique Medianero, José Luis Nieves-Aldrey

**Affiliations:** 1 Programa Centroamericano de Maestría en Entomología, Vicerrectoría de Investigación y Postgrado, Universidad de Panamá; 2 Museo Nacional de Ciencias Naturales (CSIC), Departamento de Biodiversidad y Biología Evolutiva, C/ José Gutiérrez Abascal 2, ES-28006 Madrid, Spain

**Keywords:** Cynipidae, *Barucynips*, *Coffeikokkos*, oak gall wasps, *Quercus*, Chiriqui, Volcán Barú, Panama

## Abstract

*Barucynips panamensis* Medianero & Nieves-Aldrey, a new genus and species of oak gallwasps (Hymenoptera: Cynipidae: Cynipini), is described from adults reared from galls on *Quercus bumelioides* in Panama. The new genus is taxonomically close to the recently described *Coffeikokkos* from Costa Rica, but differs from it and all of the described genera of Cynipini, by the shape and setation of the projecting part of the ventral spine of the hypopygium and by the sculpture of the propodeum. A new species of *Coffeikokkos* is also described from the same area, the Volcán Barú in Panama. Diagnostic characters, gall description, distribution, and biological data of the new genus and the two new species are given. The new genus is the first genus of oak gallwasps of the tribe Cynipini described in Panama.

## Introduction

Gall wasps (Hymenoptera, Cynipidae) are part of a quirky family inside Cynipoidea, with a majority of species being highly specialized phytophages that are able to induce complex galls on plants, although the family also contains representatives that inhabit the galls induced by other cynipids (Nieves-Aldrey 2001, Melika 2006, Liljeblad et al. 2011). The known biological diversity of the Cynipidae has recently been enlarged with the discovery that the family also includes parasitoids or lethal inquilines of gall-inducing chalcids of the family Pteromalidae on *Nothofagus* species (Nieves-Aldrey et al. 2009). The family Cynipidae is divided into eight tribes: Aylacini (157 species), Diplolepidini (58 species), Eschatocerini (3 species), Cynipini (936 species), Pediaspidini (2 species), Paraulacini (6 species), Synergini (176 species) and the recently described tribe Qwaqwaiini (1 species) (Liljeblad et al. 2011).The oak gall wasps (tribe Cynipini) is the most species-rich group of cynipid gall wasps, with the majority of its species distributed in the Holarctic region (Nieves-Aldrey 2001, Csóka et al. 2005, Liljeblad et al. 2008), although recent studies have also found a rich diversity of those insects in the Neotropical and Oriental regions (Medianero and Nieves-Aldrey 2011a, b, Tang et al. 2011).

Morphological analyses support the monophyly of the tribe Cynipini, grouping it together with the Diplolepidini, Eschatocerini and Pediaspidini into a large group of cynipid gall-inducers restricted to woody representatives of the eudicot subclass Rosidae (Kinsey 1920, Liljeblad and Ronquist 1998, Liljeblad et al. 2008). Recent molecular analyses, while confirming the monophyly of the Cynipini, failed to support the grouping with the above-mentioned tribes (Nylander 2004). Taxonomic and classificatory problems still exist in the study of the Cynipini, particularly with regard to a sound and consistent definition of the genera, especially from the rich Nearctic fauna and from the also rich, but poorly studied, neotropical and oriental faunas. The use of few and sometime inconsistent morphological characters for the separation of Cynipini genera, especially with reference to some Nearctic genera of that tribe, have often caused unstability in the classification within this tribe. Morphological studies suggest that characteristics such as the shape of the ventral spine of the hypopygium and its pilosity, the shape and sculturing of the mesosoma, the structure of the propodeum and the shape of the radial cell and Rs of the forewing can have significant potential for distinguishing genera or groups of genera (Melika and Abrahamson 2000). Other characteristics—such as the width of the head relative to the mesosoma, presence or absence of a malar sulcus, and presence, shape, depth and sculpture of scutellar foveae—are useful to complement the generic location of the species.

Recent studies have unveiled a rich diversity of Cynipidae in the high mountains of western Panama (see Medianero and Nieves-Aldrey 2011b for a synthesis). However, much of this fauna requires a more thorough taxonomic study. In line with this need, the objective of this work was to describe a new genus and species of Cynipini (Cynipidae) of the oak gall wasp fauna of Panama as well as a second species of the recently described genus *Coffeikokkos*. The new genus represents the first endemic genus of oak gall wasps in Panama.

## Materials and methods

**Study material.** The adults studied were reared from galls collected on Q*uercus bumelioides* Liebm. Samplings were made and material was collected from December 2007 to August 2010 at Volcán Barú, Chiriqui Province, Panama. The adult insects emerged from the galls in rearing cages under laboratory conditions. Voucher specimens and their galls were deposited in the entomology collections of the Museo Nacional de Ciencias Naturales, Madrid (Spain) and Maestria en Entomologia, Universidad de Panama (MEUP). The identification of the *Quercus* species was based on several key references (Burger 1977, D’Arcy 1987, Breedlove 2001) as well as on comparison with materials from the collection of the University of Panama and the Smithsonian Tropical Research Institute.

**Specimen preparation.** For observation under a scanning electron microscope (SEM), adult cynipids were dissected in 70% ethanol, air dried, mounted on a stub and coated with gold. Micrographs were taken with an FEI QUANTA 200 microscope (high vacuum technique) for several standardized views. Forewings were mounted in Euparal on slides and later examined under a Wild MZ8 stereo microscope. Images of adult habitus and gall dissections were taken with a NIKON Coolpix 4500 digital camera attached to a Wild MZ8 stereo microscope. Measurements were made with a calibrated micrometer scale attached to an ocular of the light microscope. The images will be deposited in the “morphbank.com” databank. Terminology of morphological structures and abbreviations follow Ronquist and Nordlander (1989), Ronquist (1995), Nieves-Aldrey (2001), and Liljeblad et al. (2008). For cuticular sculpture we follow Harris (1979). Measurements and abbreviations used include: POL (post-ocellar distance) is the distance between the inner margins of the posterior ocelli; OOL (ocellar-ocular distance) is the distance from the outer edge of a posterior ocellus to the inner margin of the compound eye.

## Results

### Barucynips

Medianero & Nieves-Aldrey gen. n.

urn:lsid:zoobank.org:act:2B3BA216-64F7-4E32-BA90-2BEC335C6FB9

http://species-id.net/wiki/Barucynips

Figs 1 2 3 5

#### Type species.

*Barucynips panamensis* Medianero & Nieves-Aldrey, sp. n., by present designation and monotype.

#### Etymology.

From Barú (the name of the volcano in Panama where the new genus was collected) and Cynips, referring to Cynips-groups, inside the tribe Cynipini.

#### Gender.

Masculine

#### Diagnosis and identification.

**Figure 1.**
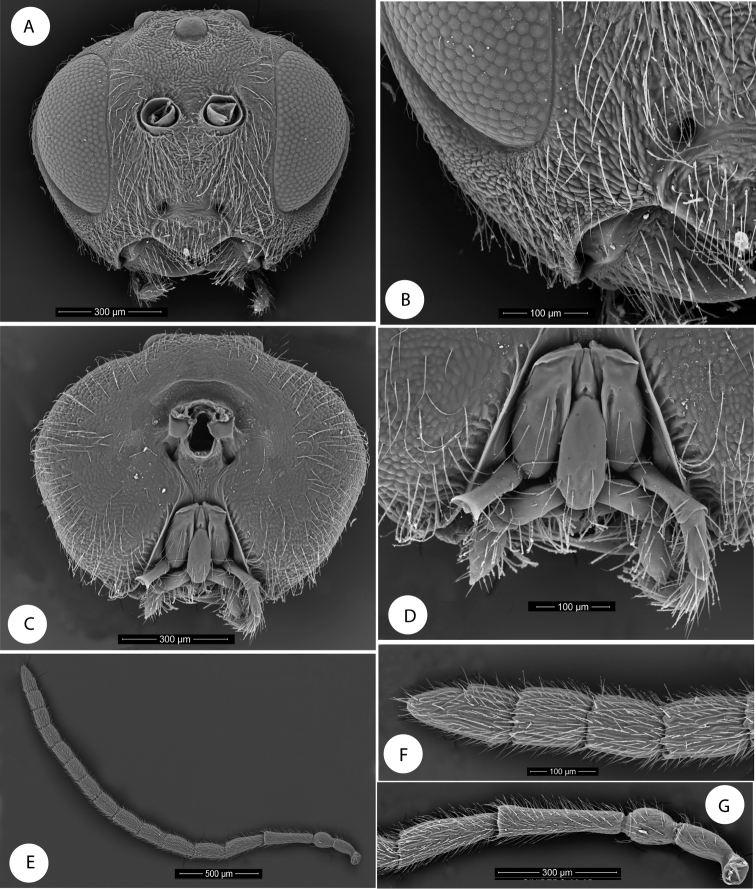
*Barucynips panamensis*: **A** Head anterior view **B** Detail of malar space **C** Head posterior view **D** Mouthparts **E** Female antenna **F** Detail of last flagellomeres **G** Detail of basal flagellomeres.

**Figure 2.**
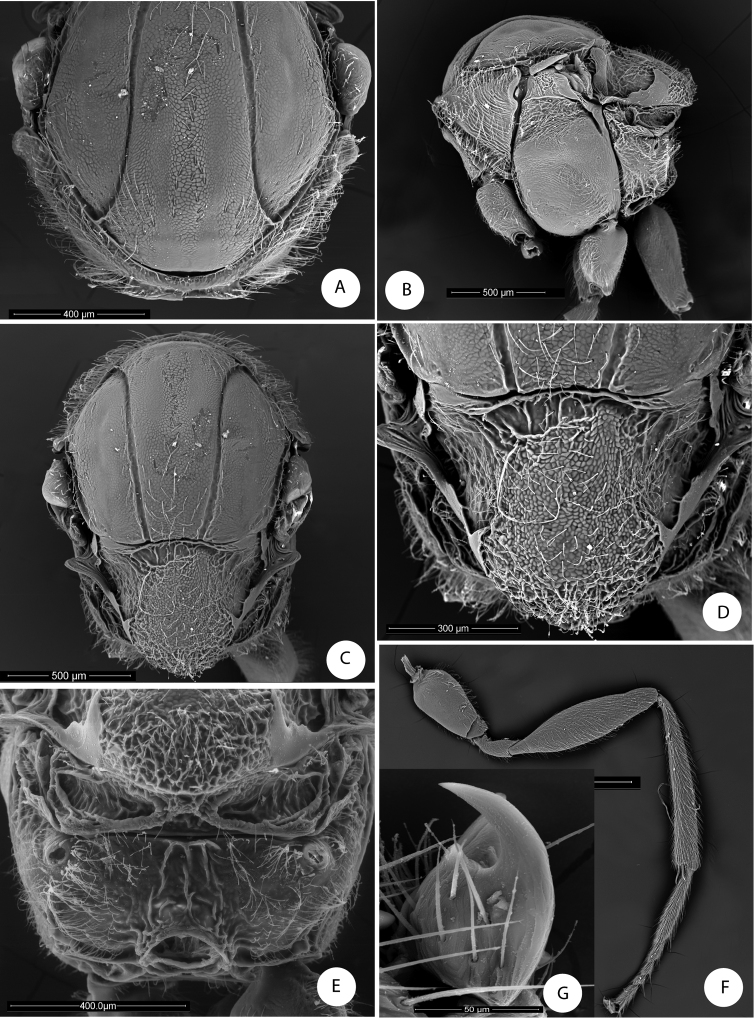
*Barucynips panamensis*: **A** Pronotum antero-dorsal view **B** Mesosoma lateral view **C** Mesosoma dorsal view **D** Scutellum **E** Propodeum **F** Hind leg **G** Metatarsal claw.

**Figure 3.**
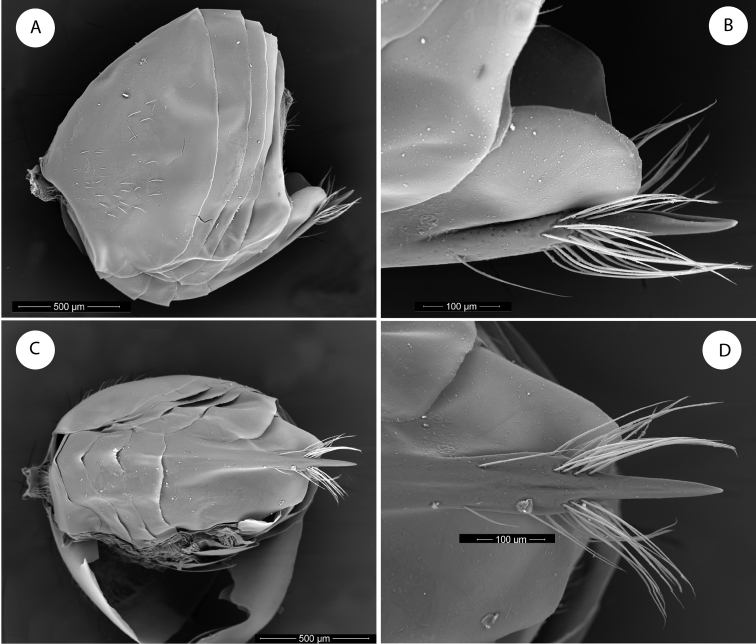
*Barucynips panamensis*: **A** Metasoma lateral view **B** Detail of ventral spine of hypopygium, lateral view **C** Metasoma ventral view **D** Detail of ventral spine of hypopygium.

By the 16 segmented antennae, head and mesosoma shape and sculpture, forewing venation and the association with *Quercus bumelioides*, a tree common in the mountains of Central America, the new genus resembles the recently described genus *Coffeikokkos* Pujade-Villar & Melika, from Costa Rica. *Barucynips* differs, however, from that genus in some important characteristics which clearly separate the two genera. The main diagnostic characteristic that clearly allows the separation of *Barucynips* and *Coffeikokkos* is the shape of the hypopygial spine. The shape and setosity of the ventral spine of hypopygium of *Barucynips* is completely distinctive among all the exhibited by the described cynipids (Fig. 3). The projecting part of the hypopygial spine is lanceolate, pointed apically, and at least 4.0 times as long as broad, with a basal group of long setae, which reach the apex of the spine, forming a tuft, while *Coffeikokkos* present a projecting part of the hypopygial spine only 2.5 times as long as wide, uniformly broad, with parallel sides, rounded distally, with tuft of long supapical setae, reaching far beyond apex of spine. The new genus also differs from *Coffeikokkos* in the propodeal sculpture. The lateral carinae of the propodeum are subparallel in the anterior half and strongly divergent posteriorly in *Coffeikokkos*, without a median longitudinal carina, while the lateral propodeal carinae are much less strongly divergent posteriorly and a median longitudinal carina is present in *Barucynips*. Furthermore, the metatarsal claws of *Coffeikokkos* present a strong basal obtuse lobe, while the claws of *Barucynips* are toothed, with a small acute basal tooth. Other discriminant characteristics are as follows: the ventral margin of clypeus is strongly projected on mandibles (only slightly so in *Coffeikokkos*); the malar area has a strong alutaceous sculpture (Fig. 1B) and lacks irradiating striae from the clypeus (a few but clearly impressed striae are present in *Coffeikokkos*). The new genus is also taxonomically closeto the genera of the *Cynips* group, *Cynips* Linnaeus (=*Antron* Kinsey, *Besbicus* Kinsey), *Atrusca* Kinsey, *Philonix* Fitch, *Acraspis* Mayr and *Biorhiza* Westwood (=*Sphaeroteras* Ashmead) but can easily be distinguished by the following character states: antenna with 16antennomeres (Fig. 1E); projecting part of the ventral spine of the hypopygium is long, lanceolate, and at least 4.0 times as long as broad; from the point where the spine narrows, a dense group of long setae arises, which reach the apex of the spine, forming a tuft, (Fig. 3B–D); lateral propodeal carinae poorly defined, fragmented, widely divergent posteriorly, with a median propodeal area bare, and narrower anteriorly, and a fragmented median longitudinal carina present (Fig. 2E); lateral propodeal area densely pubescent and forewing hyaline.

Of the genera included into the Cynips group, the new genus resemble the genus *Atrusca*, primarily because of the long ventral spine of the hypopygium, but it differs in the position, direction and length of the setae on the spine, which are subapical, reaching beyond the apex of the spine. Additionally, the species of genus *Atrusca* possesses, on the forewing, dark spots or dark stripes along veins, the radial cell is 2.0 – 2.5 times as long as broad and the Rs is strongly angulate. More diagnostic characters are given in the generic key and the description below.

#### Description.

Description of this genus is based in the asexual generation of the only known species. The eventual discovery of a sexual generation would imply the revision of the generic limits.

Asexual female densely pubescent. Head (Figs 1A–C), with genae slightly expanded behind eyes. Clypeus with ventral margin sinuate, moderately projecting over mandibles. Malar space without malar sulcus. Head, posterior view (Fig. 1C) without occipital carina. Gula short, distance between occipital and oral foramina ashigh as occipital foramen (Fig. 1C). Hypostomal sulci well separated at oral fossa.

Antenna (Fig. 1E–G) with 16 antennomeres; flagellum not broadening towards apex.

Mesosoma. Pronotum short medially, densely pubescent, lateral surface of pronotum with longitudinal wrinkles; pronotal plate indistinct dorsally (Fig. 2A). Mesoscutum alutaceus, barely pubescent with scattered setae anteriorly and posteriorly. Notauli percurrent, smooth, well impressed along entire length, reaching pronotum, separated posteriorly, with an indistinct median mesoscutal impression. Scutellar foveae not well differentiated, shallow, confluent medially, with some longitudinal striae and indistinct margins posteriorly.

Lateral propodeal carinae moderately divergent ventrally, the median propodeal area narrow, bare, with a median longitudinal carina present (Fig. 2E). Metatarsal claws with an acute basal lobe. Forewing (Fig. 5B) hyaline, without fuscate spots or stripes, radial cell open along anterior margin; areolet triangular, closed and distinct. Apical margin of wing with short hair fringe. Metasoma with second metasomal tergite covering about two thirds of metasoma, with a patch of setae laterally in its anteromedial area. Projecting part of hypopygial spine long, lance shaped, at least 4.0 times as long as broad, with dense, long basal setae that reach the apex of the spine and form a dense tuft (Fig. 3B–C).

#### Distribution.

Based on our data, the new genus is found only to 2515–3045 m a.s.l. at Volcán Barú, Chiriqui, Panama, around the upper limit of the growth of *Quercus* species in Panama.

#### Key for the identification of the genus *Barucynips* from related genera

**Table:** 

1	Antennae with 16–17 segments. Projecting part of the ventral spine of hypopygium long or short. Forewings without dark spots or stripes along veins	2
–	Antennae with 13–14 segments. Projecting part of the ventral spine of the hypopygium short, not more than 2 times as long as broad (Fig. 4A–E, 4G–H), ifprojecting part of the ventral spine of the hypopygium is long (Fig. 4F), thenthe forewing possesses dark spots and/or dark stripes along veins and the Rs is strongly angulate; setae on the spine are subapical, reaching beyond the apex of the spine	genera *Cynips*, (*=Antron, Besbicus*),* Philonix, Acraspis, Biorhiza* (*=Sphaeroteras*),* Kinseyella and Atrusca*
2	Projecting part of hypopygial spine long, lanceolate, at least 4.0 times as long as broad, with dense long basal setae that reach the apex of the spine forming a tuft (Fig. 3B–C). Female antenna with 16 segments (Fig 1E). Metatarsal claws toothed, with a small basal lobe or tooth, 1/3 as long as main tooth. Median propodeal carina present. Malar area without irradiating striae	*Barucynips*
–	Projecting part of the hypopigial spine short, at most 2.5 times as long as wide, uniformly broad, with tuft of long supapical setae reaching far beyond apex of spine. Female antenna with 17 segments. Metatarsal claws simple, with an obtuse rounded basal lobe. Median propodeal carina absent. Malar area with some irradiating striae	*Coffeikokkos*

### Barucynips panamensis

Medianero & Nieves-Aldrey sp. n.

urn:lsid:zoobank.org:act:D7878DF3-7F9C-417F-B3A0-B835AA5FE518

http://species-id.net/wiki/Barucynips_panamensis

Figs 1 2 3 5

#### Type material.

Holotype ♀ (Fig. 5A) (in Museo Nacional de Ciencias Naturales, Madrid, Spain (MNCN), card-mounted. Cat. nº 2315). PANAMA, Chiriquí, Volcán Barú 8°46'36.8"N, 82°31'39.3"W, 2515–3045 m; from galls on leaves of *Quercus bumelioides* Liebm. (Fagaceae), gall collected 16.vi.2008, insect emerged vii.08, E. Medianero leg. Paratypes: 1♀ same data as holotype, 2♀ same data as holotype but collected 15.iii.2008, insect emerged iv.08, 1♀ same data as holotype but collected 24 i.2009, insect emerged ii.09. Three paratypes in MNCN, one paratype in Maestría en Entomología, Universidad de Panamá (MEUP).

Additionally, 1♀ paratype of the type series was dissected for SEM observation (in MNCN).

#### Etymology.

Named after the country where the new species was collected.

#### Description.

Asexual female**.** Body length 2.9 mm (range 2.59–3.42; N = 4). Head and mesosoma black. Metasoma, flagellomeres and tarsi brownish; hypopygium yellowish. Mandibles yellowish with black teeth. Antennal scape and pedicel yellowish in part. Half basal of coxae black; coxae apically, femora and tibiae ventrally yellowish. Forewing hyaline, slightly and uniformly darkened; veins dark brown.

Head alutaceous-reticulate, moderately pubescent, with relatively long white setae, except on vertex, upper frons and gena, in dorsal view about 2.7 times wider than long. POL 1.8 times longer than OOL, posterior ocellus separated from inner orbit of eye by 2.0 times its longest diameter. Head in anterior view (Fig. 1A) 1.2 times wider than high. Genae slightly expanded behind eyes, strongly alutaceus-reticulate (Fig. 1B). Clypeus more or less trapezoidal, 1.7 times wider than high moderately pubescent, ventral margin sinuate, moderately projecting over mandibles. Anterior tentorial pits visible; epistomal sulcus not visible, clypeo-pleurostomal lines visible. Malar space 0.2 times height of compound eye, without malar sulcus strongly alutaceus-reticulate, without irradiating striae from clypeus (Fig. 1B). Toruli situated slightly above mid-height of compound eye; distance between antennal rim and compound eye 0.8 times width of antennal socket including rim. Ocellar plate slightly raised. Head, posterior view (Fig. 1C) without occipital carina. Gula short; distance between occipital and oral foramina ashigh as occipital foramen (Fig. 1C). Hypostomal sulci well separate at oral fossa.

Mouthparts (Figs 1D): mandibles exposed; with dense setae in base, right mandible with three teeth; left with two teeth. Cardo of maxilla visible, maxillary stipes long, about 2.3 times longer than wide. Maxillary palp five-segmented. Labial palp three-segmented, both moderately pubescent (Fig. 1D).

Antenna (Fig. 1E–G) of moderate length, as long as 1/2 body length, with 16 antennomeres; flagellum not broadening towards apex; with relatively long, erect setae, and visible elongate placodeal sensilla (Fig. 1F). Relative lengths of antennal segments: 19:12:32:25:20:21:17:18:16:15:13:12:13:12:11:16. Pedicel, globose, 0.6 as long as scape (Fig. 1G). F1 1.3 times as long as F2. F14 2.1 times longer than wide, 1.5 times as long as F13 (Fig. 1F). Placodeal sensillae on F2-F14 disposed in one row of 8–9 sensillae in half dorsal area of each flagellomere.

Mesosoma. Uniformlyalutaceus, moderately pubescent, in lateral view 1.2 times as long as high, slightly convex dorsally (Fig. 2B). Pronotum, densely pubescent; lateral surface of pronotum with longitudinal wrinkles; with long and dense white setae (Fig. 2A-B). Pronotum short medially, ratio of length of pronotum medially/laterally = 0.2. Pronotal plate indistinct dorsally (Fig. 2A).

Mesonotum (Fig. 2C). Mesoscutum coriaceous-alutaceus, only slightly pubescent medially and along notauli, slightly broader than long in dorsal view. Notauli percurrent, smooth, well impressed along entire length, reaching pronotum, not quite convergent, well separated posteriorly, median mesoscutal impression not visible. Anteroadmedian and parapsidal signa visible. Transscutal fissure narrow, well-visible, deeply impressed, slightly sinuate. Scutellar foveae not well differentiated, shallow, confluent medially, with some longitudinal striae and indistinctly margined posteriorly. Mesoscutellum (Fig. 2D) rounded from above moderately pubescent, about 0.6 as long as mesoscutum, strongly alutaceus-reticulate, in lateral view extending posteriorly slightly over the dorsellum. Axillula slightly pubescent, their anterior margins marked and posterior indistinctly. Mesopleuron alutaceus, slightly pubescent with mesopleural triangle densely pubescent (Fig. 2B).

Metanotum (Fig. 2E). Metapectal-propodeal complex. Metapleural sulcus reaching posterior margin of mesopectus at about two thirds height (Fig. 2B). Lateral propodeal carinae poorly defined, fragmented, slightly divergent posteriorly and reaching nucha. Median propodeal area narrow, bare, with a median longitudinal carina fragmented but well visible (Fig. 2E); lateral propodeal area densely pubescent, with relatively long white setae, nucha rugose.

Legs (Fig. 2F). Densely pubescent, metatarsal claws with an acute basal lobe or short tooth (Fig. 2G).

Forewing (Fig. 5B) 1.2 times as long as body, without smoky spots or stripes and densely pubescent; basal cell pubescent; radial cell 4.2 times longer than wide, open along anterior margin; areolet large, triangular, closed. R1, Rs and M nearly straight not reaching wing margin. R1 forming a quite acute angle with anterior margin of wing; Rs+M not reaching basalis. Basalis slightly curved, 2r well pigmented. Apical margin of wing with moderately long hair fringe.

Metasoma (Fig. 3A) large, as long as head and mesosoma combined, in lateral view as wide as high. Second metasomal tergite covering about two thirds of metasoma, with a patch of setae in its lateral anteromedial area. Projecting part of hypopygial spine long, lanceolate, tapering from the base to apex, at least 4.0 times as long as broad, with dense long basal setae arising in the base of the projected part which reach the apex of the spine forming a tuft (Fig. 3B–C).

**Gall** (Fig. 5C–G). Irregular small formations (up to 4–9 mm) arising from crevices on the stems and on the petiole and midribs of leaves. The gall is displayed as a dense mass covered with light brown hairs, solitary, containing a single larval cell or more frequently forming clusters, and then appearing as polythalamous. Inside, the gall has a highly lignified core enclosing the larva (Fig. 5E).

**Host plant**. *Quercus bumelioides* Liebm. (section Quercus of *Quercus*; white oaks (Fagaceae), a species distributed from Mexico to Panama (Breedlove 2001). The gall resembles that of the species of the Nubila complex, established by Kinsey within the subgenus *Acraspis* of *Cynips* (Kinsey 1936), all known from Mexico. These galls were described as a mass of coarse hairs containing a spherical hard core, attached to mid-veins, on or under the surfaces of leaves. However, the insects are quite different in important characteristics such as the number of antennal segments and the shape of the hypopygial spine.

#### Distribution.

*Barucynips panamensis* was found between 2515–3045 m a.s.l. at Volcán Barú, Chiriqui, Panama. Although currently known only at this locality, it is a species that is relatively abundant at the higher elevations of Volcán Barú.

#### Biology.

Only the asexual generation is known, inducing galls on *Quercus bumelioides* Liebm (section Quercus). The galls are common and can be found at every time of year in different grades of maturation on stems and leaves. The galls frequently are found growing together with galls of the new species, also described in this paper, *Coffeikokkos korytkowskii*. The insects studied emerged from January to July.

#### Inquiline and parasitoid associated community.

From the gall of *Barucynips panamensis*, two species of inquilines were reared, *Synergus elegans* Nieves-Aldrey & Medianero, and one indeterminate species similar to *Synergus luteus* Nieves-Aldrey & Medianero or *Synergus gabrieli* Nieves-Aldrey & Medianero (Nieves-Aldrey and Medianero 2011). Additionally, the parasitoid species *Ormyrus venustus* Hanson 1992 (Chalcidoidea, Ormyridae) was also reared.

**Figure 4.**
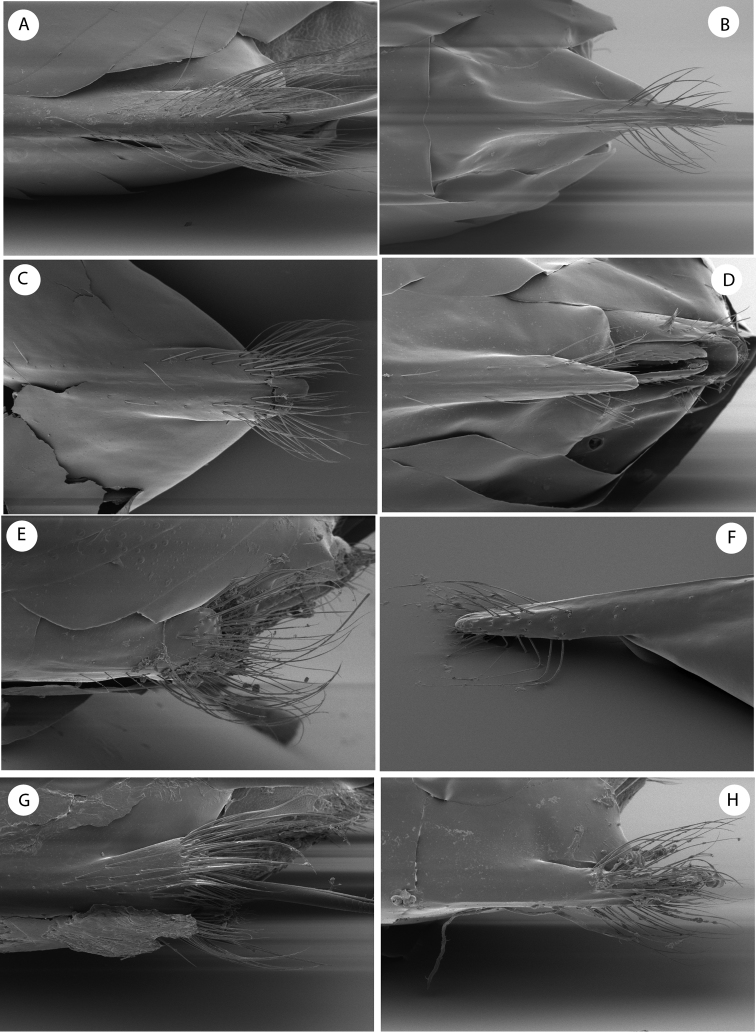
Detail of ventral spine of hypopygium of females of species of the Cynips group: **A** *Cynips divisa*, asexual generation **B** *Cynips divisa*, sexual generation **C** *Acraspis erinacei*, asexual generation **D** *Acraspis erinacei*, sexual generation **E** *Cynips* =*Antron douglasi* **F** *Atrusca emergens* **G** *Philonix gigas* **H** *Biorhiza* =*Sphaeroteras mellea*. [after Liljeblad *et al*. 2008 http://www.morphbank.net/].

**Figure 5.**
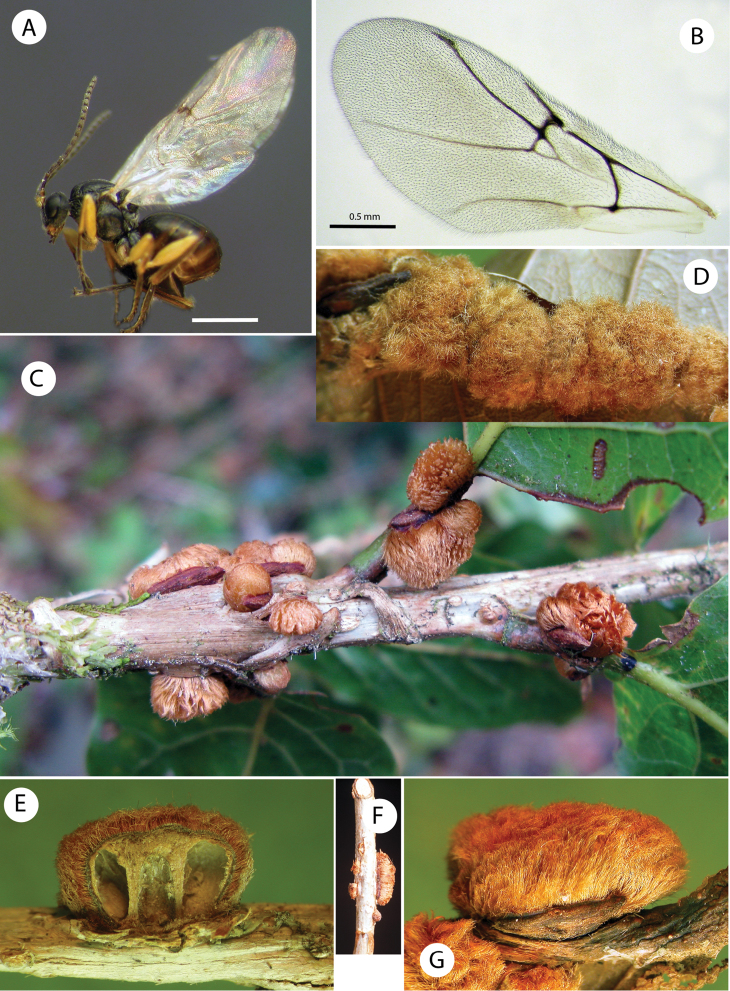
Habitus, forewings and galls of *Barucynips panamensis* **A** Habitus, female **B** Forewing of female **C–G** Mature galls **C** Galls on stems **D** Cluster of galls on midrib of leaves **E** Section of a gall showing larval cells **F** Galls on stems **G** Detail of a single gall.

**Figure 6.**
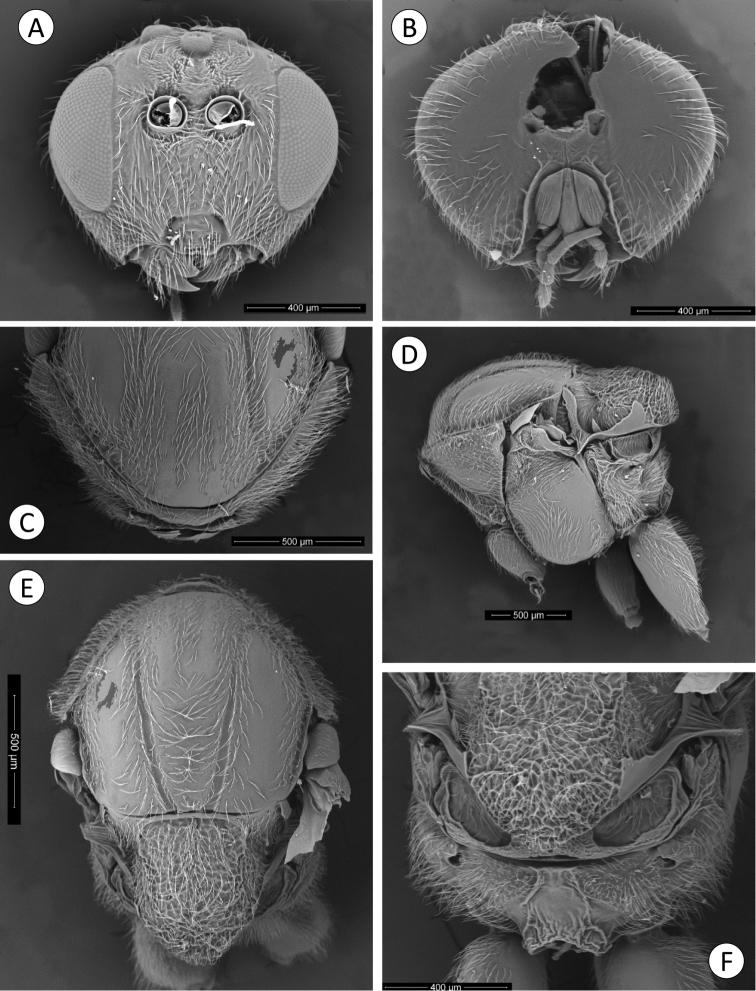
*Coffeikokkos korytkowskii*: **A** Head anterior view **B** Head posterior view **C** Pronotum antero-dorsal view **D** Mesosoma lateral view **E** Mesosoma dorsal view **D** Propodeum.

### Coffeikokkos korytkowskii

Medianero & Nieves-Aldrey sp. n.

urn:lsid:zoobank.org:act:D8B6DD27-E6CB-44A4-9598-BBCD51D7E3F5

http://species-id.net/wiki/Coffeikokkos_korytkowskii

Figs 6 [Fig F7] –8

#### Type material.

Holotype ♀ (Fig. 8A) (in Museo Nacional de Ciencias Naturales, Madrid, Spain (MNCN), card-mounted. Cat. nº 2316). PANAMA, Chiriquí, Volcán Barú 8°46'36.8"N, 82°31'39.3"W, 3079 m; ex gall on stems of *Quercus bumelioides* Liebm. (Fagaceae), gall collected 23.x.2008, insect emerged xi.08, E. Medianero leg. Paratypes: 12♀ same data as holotype. Seven paratypes in MNCN, five paratypes in Maestría en Entomología, Universidad de Panamá (MEUP).

Additionally, 1♀ paratype of the type series was dissected for SEM observation (in MNCN).

#### Etymology.

Named after Dr. Cheslavo A. Korytkowski for his contribution to the development of entomology in Panama.

#### Diagnosis and comments.

The new species represents the second species of the genus *Coffeikokkos*, which was recently described in Costa Rica. The species is closely related to *Coffeikokkos copeyensis* Pujade-Villar & Melika, 2012, being similar in color and a majority of morphological characteristics. The species differ in the pubescence of mesosoma, the shape of the mesoscutellum, propodeal sculpture and gall shape. *Coffeikokkos korytkowskii* has a moderately pubescent mesosoma with piliferous punctures, whereas the mesosoma is smooth in *Coffeikokkos copeyensis*. The new species has a more elongate mesoscutellum, clearly longer than is wide, and the propodeal carinae are complete, reaching the nucha, whereas in *Coffeikokkos copeyensis*, the mesoscutellum is as long as is wide or only slightly longer than wide, and the propodeal carinae is incomplete, not reaching the nucha. Additionally, the basal cell of the forewing in the new speciesis hairy, whereas the basal cell of *Coffeikokkos copeyensis* is bare. The new species induces a regular spherical gall (5 mm diameter) with a spotty surface, while the galls induced by *Coffeikokkos copeyensis* are similar in size, but irregular or slightly ovate and uniformly colored.

#### Description.

Body length 3.62 mm (range 3.3–4.2; N = 5) for females. Body uniformly reddish brown and shiny; the toruli area, flagellomeres of antenna, area above clypeus, occiput, dorsolateral margin of pronotum, anteroadmedian signa area, parapsidal signa, mesopleuron, metapectal-propodeal complex and anteromedial area of scutellum dark brown to black. Legs with all coxae and femora yellowish; tibia and tarsomeres dark brown to black. Forewing hyaline with some very light infumation; veins dark brown to black.

*Asexual female*. Head moderately pubescent with piliferous punctures, in dorsal view about 3.5 times wider than long. POL 1.5 as long as OOL, posterior ocellus separated from inner orbit of eye by 1.7 times its longest diameter. Head in anterior view (Fig. 6A) transversely ovate, 1.15 times wider than high, gena not expanded behind eyes. Vertex frons and gena slightly alutaceous. Head moderately pubescent, with relatively long white setae, except vertex, frons with sparse, shorter setae. Clypeus more or less trapezoidal, 1.6 times wider than high, mostly smooth and moderately pubescent; ventral margin sinuate, slightly projecting over mandibles. Anterior tentorial pits visible; epistomal sulcus indicated, clypeo-pleurostomal lines visible. Malar space 0.3 times height of compound eye, without malar sulcus; some irradiating striae from clypeus present, reaching ventral margin of compound eye, absent medially above clypeus. Toruli situated slightly above mid-height of compound eye; distance between antennal rim and compound eye 1.1 times width of antennal socket including rim. Ocellar plate not raised. Head, posterior view (Fig. 6B) without occipital carina. Gula short; distance between occipital and oral foramina 0.5 times height of occipital foramen (Fig. 6B). Hypostomal sulci well separate at oral fossa.

Mouthparts (Figs 6A–B): mandibles exposed, with dense setae in base, right mandible with three teeth, left with two teeth. Cardo of maxilla not visible, maxillary stipes about 2.3 times longer than wide. Maxillary palp five-segmented. Labial palp three-segmented.

**Figure 7. F7:**
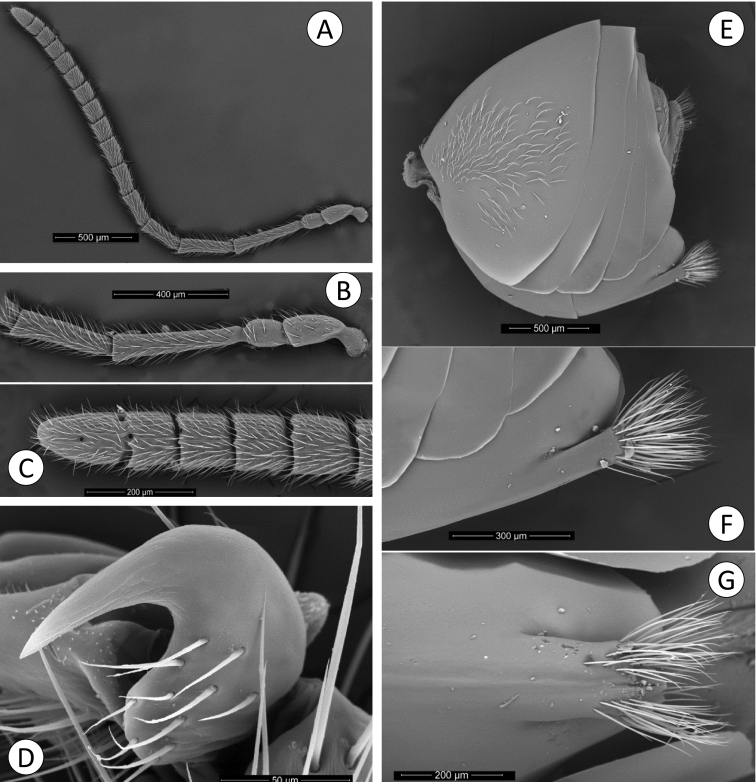
*Coffeikokkos korytkowskii*: **A** Female antenna **B** Detail of basal flagellomeres **C** Detail of last flagellomeres **D** Metatarsal claw **E** Metasoma lateral view **F** Detail of ventral spine of hypopygium, lateral view **G** Detail of ventral spine of hypopygium.

Antenna (Fig. 7A-C) of moderate length, as long as 1/2 body length, with 15 flagellomeres, but F15 only partially separated from F14 dorsally (Fig. 7C); flagellum not broadening towards apex; with relatively long, erect setae, and elongate placodeal sensilla well visible (Fig. 7C). Relative lengths of antennal segments: 16:12:43:32:24:20:19:17:13:12:11:11:10:9:9:8:14. Pedicel sub-globose, 0.8 as long as scape; F1-F9, gradually decreasing in length. F1 1.34 times as long as F2. F10-F13 short and wide, F15 1.8 times longer than wide, 1.7 as long as F14 (Fig. 7C). Placodeal sensillae on F4-F15 disposed in one row of 5–6 sensillae in half dorsal area of each flagellomere.

Mesosoma. Smooth, moderately pubescent with piliferous punctures, in lateral view 1.3 times as long as high, slightly convex dorsally. Pronotum, moderately pubescent; lateral surface of pronotum with some longitudinal wrinkles dorsally; with long and dense setae (Fig. 6D). Pronotum short medially, ratio of length of pronotum medially/laterally = 0.16. Pronotal plate indistinct dorsally (Fig. 6C).

Mesonotum (Fig. 6E). Mesoscutum smooth, moderately pubescent with piliferous punctures medially and along notauli; slightly broader than long in dorsal view. Notauli complete, smooth, broad, deep and convergent posteriorly, without median mesoscutal impression, anteroadmedian signa and parapsidal signa indistinct. Transscutal fissure narrow, clearly visible, deeply impressed, and slightly sinuate. Scutellar foveae shallow, confluent, indistinctly margined posteriorly and rugose. Mesoscutellum (Fig. 6E), about 0.7 times length of mesoscutum, 1.2 times as long as wide, strongly reticulate-rugose and moderately pubescent, in lateral view extended posteriorly over dorsellum. Axillula moderately pubescent, their anterior margins marked and posterior margins indistinct. Mesopleuron smooth, moderately pubescent except in speculum (Fig. 6D).

Metanotum (Fig. 6F). Metapectal-propodeal complex. Metapleural sulcus reaching posterior margin of mesopectus at about mid-height of metapectal-propodeal complex (Fig. 6D). Lateral propodeal carinae moderately divergent posteriorly, reaching the nucha; median propodeal area longer than broad, smooth, with some setae anteriorly; lateral propodeal area densely pubescent (Fig. 6F). Nucha rugose.

Legs. Densely pubescent; metatarsal claws with a large obtuse basal lobe (Fig. 7D).

**Figure 8.**
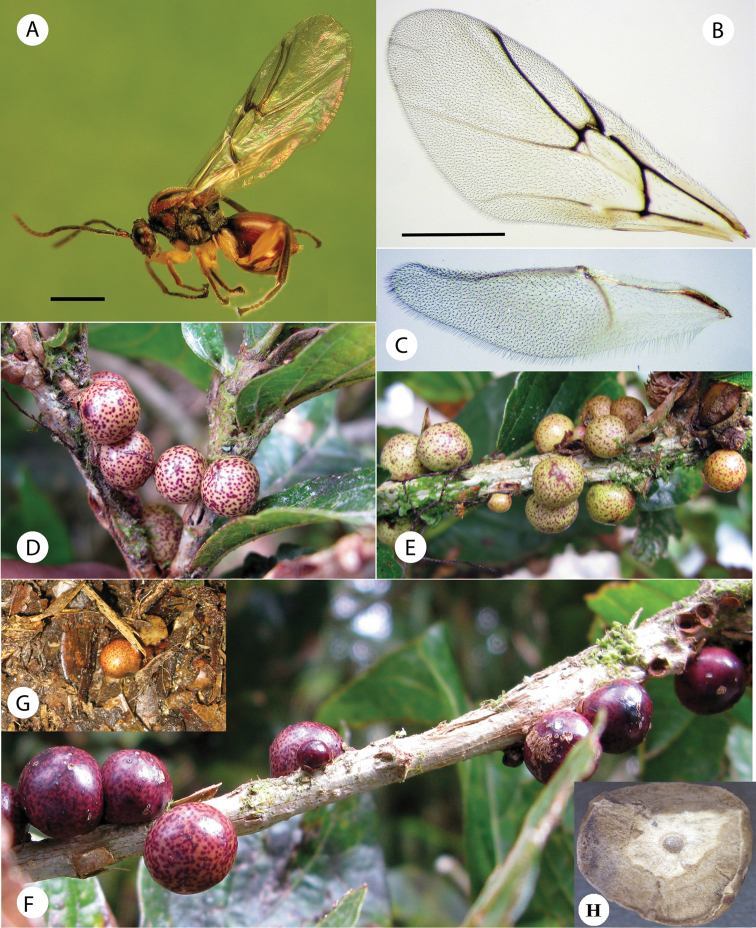
*Coffeikokkos korytkowskii*: **A** Habitus, female **B** Fore wing **C** Hind wing **D–F** Galls **G** A gall on the ground **H** Section of a gall showing larval cells.

Forewing (Fig. 8B) slightly longer than body, strongly pubescent; basal cell with some rows of setae; radial cell 4.0 times longer than wide; open along anterior margin; areolet triangular, closed and distinct. R1 and Rs nearly straight, not quite reaching wing margin; R1 forming an acute angle with anterior margin of wing. Rs+M reaching basalis at its mid-height. 2r well pigmented, angulate and slightly projected medially. Apical margin of wing with moderately long hair fringe.

Metasoma (Fig. 7E) large, as long as head and mesosoma combined, in lateral view as wide as high. Second metasomal tergite covering about 2/3 of metasoma, with a patch of dense setae in its anteromedial area. Projecting part of hypopygial spine short (Fig. 7F–G), shorter than basal height of spine (Fig. 7F); with parallel sides and pointed apically, with dense long subapical setae forming a patch, extending far beyond apex of spine.

***Gall*** (Fig. 8D–H). Similar in location, shape, and size to the galls of *Coffeikokkos copeyensis* Pujade-Villar & Melika. However, the galls of this new species are much more regularly spherical and its surface is not uniformly colored, but spotty. Diameter of gall measures 5 to 8 mm. They are formed, solitary or more frequently in groups, in stems of *Quercus bumelioides* Liebm. The surface of the gall is smooth and shiny; whitish, green or yellowish when fresh with red spots, becoming brown when mature. Monothalamic, with compact woody tissue internally containing the single larval cell (Fig. 8H). Similar spherical and spotty galls are also induced by the Nearctic *Cynips* (=*Besbicus*) *mirabilis* (Kinsey 1922), but this galls are larger, pubescent, formed in leaves and with an internal structure of irradiant filaments (Kinsey 1930).

#### Distribution.

*Coffeikokkos korytkowskii* was found between 2515–3045 m a.s.l. at Volcán Barú, Chiriqui, Panama.

#### Biology.

Only the asexual generation is known, inducing galls on *Quercus bumelioides* Liebm. (section *Quercus*). The galls are common and can be found at every time of year in different grades of maturation on stems. They often develop jointly with the galls of *Barucynips panamensis*, also described here. When the gall is mature, it falls to the ground (Fig. 8G), from which the adult emerges after a couple of months. The studied insects emerged in November.

#### Key for the identification of species of genus *Coffeikokkos*

**Table:** 

1	Temples only slightly expanded behind eyes. Mesosoma conspicuously pubescent; mesoscutum medially setose with piliferous punctures. Mesoscutellum elongate, longer than wide. Propodeal carinae moderately divergent posteriorly, complete, reaching the nucha. Basal cell of forewing with some setae. Areolet small, measuring less than 1/3 length of Rs+M. Gall regularly spherical, with colored spotty surface	*Coffeikokkos korytkowskii*
–	Temples more clearly expanded behind eyes, being visible in anterior view of head. Mesosoma slightly pubescent; mesoscutum medially smooth, shiny, with only sparse setae laterally and along notauli. Mesoscutellum as long as wide. Propodeal carinae strongly divergent posteriorly, incomplete not reaching the nucha. Basal cell of forewing almost bare. Areolet large, measuring about ½ of length of Rs+M. Galls irregular, with uniform red colour, without colored spots	*Coffeikokkos copeyensis*

## Discussion

The majority of the morphological characters of *Coffeikokkos* and the new proposed genus *Barucynips* indicate their close affinity with the “*Cynips* group” of genera (Kinsey 1930, 1936, Liljeblad et al. 2008). Their association only with *Quercus bumelioides*, a species of “white oaks” (*Quercus* section Quercus), reinforce the morphological evidence. The genera of the Cynips group, which is represented primarily in the Nearctic Region, have had an unstable taxonomic status. Kinsey (1930, 1936), in his revision of the genus *Cynips*, divided it into six subgenera: *Cynips* (European species) *Antron*, *Besbicus*, *Atrusca*, *Philonix*, and *Acraspis* (American species). Under the “mellea” species complex in the *Acraspis* subgenus, he included also all known *Sphaeroteras* species (Melika and Abrahamson 2002). Weld (1952) gave the status of genus to all subgenera of Kinsey, but Melika and Abrahamson (2002) consider *Antron* and *Besbicus* synonyms of *Cynips*, and *Sphaeroteras* a synonym of *Biorhiza*. Liljeblad et al. (2008) in one analyses of 308 characters, 283 from morphology and 25 from biology and distribution, suggested that these last synonymyzations were unfortunate, as they showed the close and unresolved phylogenetic relationships between the taxa forming the *Cynips* group, including the *Trigonaspis,* *Belocnonema* and *Biorhiza* species. However, because of the relatively poor taxon sampling in their analysis and the lack of convincing support values for the clades within the Cynips group, they refrained from proposing formal taxonomic changes in Cynips group. Pujade-Villar et al. (2010) described one new genus from Mexico (*Kinseyella*) related to Cynips group and suggested that the neartic genera *Antron* and *Besbicus* were erroneously synonymized to *Cynips* by Melika and Abrahamson (2002).

Despite the unstable taxonomic status of this group of genera and the clear need for a complete revision, mainly in the Neartic fauna, the new genus proposed herein, as with the recently described *Coffeikokkos*, present clearly distinctive morphological characters that justify its establishment. Both genera are closely related and share a unique synapomorphy within the Cynipini, namely, the antenna with 14–15 flagellomeres. However, *Barucynips* is well distinguished from *Coffeikokkos* and from all known Cynipini genera by one distinctive morphological character, the shape and pubescence of the projecting part of the ventral spine of the hypopygium. Furthermore, a combination of other morphological character, such as the presence of a median propodeal carina and a weakly toothed metatarsal claw lend further support to the status of this proposed new genus. In Panama, we have found more species clearly included in the Cynips group of taxa but that are doubtfully assigned to any described genus. Future studies should clarify whether these species should be attributed to the genus *Cynips* (*sensu lato*) or to one or more new genera. These facts clearly show the taxonomic complexity of this fauna and note the necessity of more complete revisional studies.

The southern boundaries of distribution of the oak gall wasps in America (the Cynipidae associated with Fagaceae) are revealing an unexpectedly great taxonomic richness and phylogenetic diversity. As evidenced by gall diversity in Panama alone, we have demonstrated that a rich fauna of 45–65 species of Cynipidae do exist in this country, contrasting with the single species previously recorded (Nieves-Aldrey and Medianero 2011, Medianero and Nieves-Aldrey 2011b). Furthermore, many genera previously known only in the Nearctic region, such as *Amphibolips*, *Disholcaspis*, *Odontocynips*, *Bassetia*, and *Loxaulus*, have been found in recent years in the Neotropical region from Costa Rica to Colombia (Medianero and Nieves-Aldrey 2010a, 2010b, 2011a, 2011b, Medianero et al. 2011a, 2011b), while representatives of undescribed species related to *Neuroterus*, *Dryocosmus*, *Cynips*, *Trigonaspis* and *Callirhytis* have also been found but have been not yet published (Medianero and Nieves-Aldrey unpub.). More interesting is the discovery of new genera such as *Agastoroxenia* (Nieves-Aldrey and Medianero 2010) from Panama, *Coffeikokkos* (Pujade-Villar et al. 2012a) and *Zapatella* (Pujade et al. 2012b) from Costa Rica and Colombia, and the new genus *Barucynips* from Panama described here, demonstrating that the phylogenetic diversity is high in this region. All of these data support Kinsey’s (1936) hypothesis, which postulated America as the center of origin and radiation of the Cynipini, and challenges the more recent hypothesis that postulates that gall wasp lineages diverged in Asia (Stone et al. 2008). New data collected in the Eastern Palearctic and oriental biogeographic region, as well as ongoing phylogenetic and biogeographical studies, will shed more light on this problem. Current data on the richness and diversity of oak gall wasps in the the Neotropic clearly indicate that this fauna reflects the result of the population dynamics along Pleistocene glaciations (circa 2.0 million to 12,000 years before present), with the high peaks of Neotropical mountains playing the role of postglacial refugees for the gall wasp fauna and their host plants.

## Supplementary Material

XML Treatment for Barucynips

XML Treatment for Barucynips panamensis

XML Treatment for Coffeikokkos korytkowskii

## References

[B1] Burger W (1977) Fagaceae. In: Burger W (Ed.). Flora Costaricensis. Fieldiana: Botany 40: 59–82.

[B2] Breedlove D (2001) Fagaceae. In: Stevens WD Ulloa UC Pool A Montiel OM (Eds). Flora de Nicaragua. Monographs in Systematic Botany. Missouri Botanical Garden 85(2): 1076-1084.

[B3] Csóka G Stone GN Melika G (2005) Biology, Ecology and Evolution of gall-inducing Cynipidae. In: Raman A Schaefer CW Withers TM (Eds). Biology, ecology and evolution of gall-inducing arthropods. Science Publishers, Inc. Enfield, New Hampshire: 569-636.

[B4] D’Arcy WG (1987) Flora of Panama. Checklist and Index. Part I. Monographs in Systematic Botany. Missouri Botanical Garden 18 (2): 1-672.

[B5] Harris R (1979) A glossary of surface sculpturing. State of California, Department of Food and Agriculture. Occasional Papers in Entomology 28: 1-31.

[B6] Kinsey AC (1920) Phylogeny of cynipid genera and biological characteristics. Bulletin of the American Museum of Natural History 42: 357-402.

[B7] Kinsey AC (1930) The gall wasp genus Cynips: A study in the origin of species. Indiana University Studies84–86: 1–577.

[B8] Kinsey AC (1936) The origin of the higher categories in Cynips. Indiana University, Publication of Science Series4: 1–334.

[B9] Liljeblad J Ronquist F (1998) A phylogenetic analysis of higher level gall wasp relationships (Hymenoptera: Cynipidae). Systematic Entomology 23: 229-252. doi: 10.1046/j.1365-3113.1998.00053.x

[B10] Liljeblad J Ronquist F Nieves-Aldrey JL Fontal-Cazalla F Ros-Farre P Gaitros D Pujade-Villar J (2008) A fully web-illustrated morphological phylogenetic study of relationships among oak gall wasps and their closest relatives (Hymenoptera: Cynipidae). Zootaxa 1796: 1-73.

[B11] Liljeblad J Nieves-Aldrey JL Neser F Melika G (2011) Adding another piece to the cynipoid puzzle: the description of a new tribe, genus and species of gall wasp (Hymenoptera: Cynipidae) endemic to the Republic of South Afric*a*. Zootaxa 2806: 35-52.

[B12] Medianero E Nieves-Aldrey JL (2010a) The genus *Amphibolips* Reinhard (Hymenoptera: Cynipidae: Cynipini) in the Neotropics, with description of three new species from Panama. Zootaxa 2360: 47-62.

[B13] Medianero E Nieves-Aldrey JL (2010b) Description of the first Neotropical species of *Bassettia* Ashmead (Hymenoptera: Cynipidae: Cynipini) from Panama. Graellsia 66 (2): 213-220. doi: 10.3989/graellsia.2010.v66.029

[B14] Medianero E Nieves-Aldrey JL (2011a) First record of the genus *Disholcaspis* Dalla Torre & Kieffer (Hymenoptera: Cynipidae: Cynipini) in the Neotropics, with description of two new species from Panama. Zootaxa 2802: 23-33.

[B15] Medianero E Nieves-Aldrey JL (2011b) Primer estudio de las avispas de las agallas de la República de Panamá, incluyendo una lista actualizada de los cinípidos neotropicales (Hymenoptera, Cynipoidea, Cynipidae). Boletín de la Sociedad Entomológica Aragonesa 48: 89-104.

[B16] Medianero E Nieves-Aldrey JL Melika G (2011a) Two new neotropical species of oak gall wasps of the genus *Loxaulus* Mayr (Hymenoptera: Cynipidae: Cynipini) from Panama. Zootaxa 2811: 37-46.

[B17] Medianero E Nieves-Aldrey JL Pujade-Villar J (2011b) The genus *Odontocynips* Kieffer (Hymenoptera: Cynipidae: Cynipini) in Panama, with redescription of *Cynips championi* Cameron, 1883. Graellsia 67: 35-46. doi: 10.3989/graellsia.2011.v67.033

[B18] Melika G (2006) Gall Wasps of Ukraine. Cynipidae. Vestnik zoologii, supplement 21(1/2): 1–644.

[B19] Melika G Abrahamson WG (2000) Historical review and current state of the world generic classification of oak gall wasp (Hymenoptera:Cynipidae: Cyninipi). In: Austin AD Downton M (Eds). Hymenoptera, Evolution and Biological control. CSIRO. Melbourne: 218-230.

[B20] Melika G Abrahamson WG (2002) Review of the world genera of oak cynipid wasps (Hymenoptera:Cynipidae, Cynipini). In: Melika G Thuróczy C (Eds). Parasitic Wasps: Evolution, Systematics, Biodiversity and Biological Control. Agroinform, Budapest: 150-190.

[B21] Nieves-Aldrey JL (2001) Hymenoptera, Cynipidae. In: Ramos MA Alba-Tercedor J Bellés i Ros X Gosálbez i Noguera J Guerra Sierra A Macpherson Mayol E Martín Piera F Serrano Marino J Templado González J (Eds). Fauna Ibérica. Vol. 16. Museo Nacional de Ciencias Naturales, CSIC, Madrid: 1-636.

[B22] Nieves-Aldrey JL Liljeblad J Hernández Nieves M Grez A Nylander JAA (2009) Revision and phylogenetics of the genus *Paraulax* Kieffer (Hymenoptera, Cynipidae) with biological notes and description of a new tribe, a new genus, and five new species. Zootaxa 2200: 1-40

[B23] Nieves-Aldrey JL Medianero E (2010) *Agastoroxenia panamensis*, a new genus and species of inquiline oak gallwasps (Hymenoptera: Cynipidae: Synergini) of the neotropic. Annals of the Entomological Society of America 103 (4): 492-499. doi: 10.1603/AN09148

[B24] Nieves-Aldrey JL Medianero E (2011) Taxonomy of inquilines of oak gall wasps of Panama, with description of eight new species of *Synergus* Hartig (Hymenoptera, Cynipidae, Synergini). Zootaxa 2744: 1-47.

[B25] Nylander JAA (2004) Bayesian Phylogenetics and the evolution of gall wasps. Ph.D. Thesis. Uppsala University, Sweden.

[B26] Pujade-Villar J Romero-Rangel S Chagoyán-García C Equihua-Martínez A Estrada-Venegas EG Melika G (2010) A new genus of oak gallwasps, *Kinseyella* Pujade-Villar & Melika, with a description of a new species from Mexico (Hymenoptera: Cynipidae: Cynipini). Zootaxa 2335: 16-28.

[B27] Pujade-Villar J Hanson P Melika G (2012a) A new genus of oak gallwasp, *Coffeikokkos* Pujade-Villar & Melika, gen. n., with a description of a new species from Costa Rica (Hymenoptera, Cynipidae). ZooKeys 168: 19-29. doi: 10.3897/zookeys.168.2030 PMC3293441 22423188

[B28] Pujade-Villar J Hanson P Medina CA Torres M Melika G (2012b) A new genus of oak gallwasps, *Zapatella* Pujade-Villar & Melika, gen. n., with a description of two new species from the Neotropics (Hymenoptera, Cynipidae, Cynipini). ZooKeys 210: 75-104. doi: 10.3897/zookeys.210.3014 PMC3406454 22859897

[B29] Ronquist F (1995) Phylogeny and early evolution of the Cynipoidea (Hymenoptera). Systematic Entomology 20: 309-335. doi: 10.1111/j.1365-3113.1995.tb00099.x

[B30] Ronquist F Nordlander G (1989) Skeletal morphology of an archaic cynipoid, Ibalia rufipes (Hymenoptera, Ibaliidae). Entomologica Scandinavica Supplements 33: 1-60.

[B31] Stone GN Atkinson RJ Rokas A Nieves-Aldrey JL Melika G Acs Z Csóka G Hayward A Bailey R Buckee C McVean GAT (2008) Evidence for widespread cryptic sexual generations in apparently purely asexual *Andricus gallwasps*. Molecular Ecology 17: 652-665. doi: 10.1111/j.1365-294X.2007.03573.x 18086197

[B32] Tang CT Melika G Nicholls JA Yang MM Stone GN (2011) A new genus of oak gallwasps, *Cycloneuroterus* Melika & Tang, with the description of five new species from Taiwan (Hymenoptera: Cynipidae: Cynipini). Zootaxa 3008: 33–62.

[B33] Weld LH (1952) Cynipoidea (Hym.) 1905–1950. Privately published, Ann Arbor, Michigan, 351 pp.

